# Beyond the Stent (“Leave-Nothing-Behind”) Drug-Coated Balloons in Acute Coronary Syndrome: A Narrative Review

**DOI:** 10.3390/jcm15124491

**Published:** 2026-06-10

**Authors:** Sheref Zaghloul, Ahmed Shahin, Salaheldin Agamy, Kalliopi J. Ioakim, Mohamed Aly, Luciano Candilio

**Affiliations:** 1 Cardiology Department, Royal Berkshire Hospital, Reading RG1 5AN, UK; 2 Cardiology Department, Hampshire Hospitals NHS Foundation Trust, Basingstoke RG24 9NA, UK; 3 Cardiology Department, University Hospitals Birmingham NHS Foundation Trust, Birmingham B15 2GW, UK; salaheldin.agamy@nhs.net; 4 Internal Medicine Department, Queen Elizabeth Hospital, Birmingham B15 2GW, UK; 5 Cardiology Department, University Hospitals of North Midlands NHS Trust, Stoke-on-Trent ST4 6QG, UK; mohamed.aly6@nhs.net; 6 Cardiology Department, Royal Free London NHS Foundation Trust, London NW3 2QG, UK

**Keywords:** drug-coated balloon, acute coronary syndrome, percutaneous coronary intervention, de novo lesions, artificial intelligence, robotic-assisted PCI, paclitaxel, sirolimus, in-stent restenosis

## Abstract

**Background**: Drug-coated balloons (DCBs) have emerged as a “leave-nothing-behind” strategy in percutaneous coronary intervention (PCI), with potential advantages over drug-eluting stents (DES) in selected patients with acute coronary syndrome (ACS). **Methods**: We performed a narrative review of randomized controlled trials, registries, and meta-analyses evaluating DCB therapy in ACS, including PEPCAD NSTEMI, REVELATION, BASKET-SMALL 2, AGENT IDE, REC-CAGEFREE I/II, and the ongoing TRANSFORM II trial. Articles were identified through searches of PubMed/MEDLINE, Embase, Scopus, Web of Science, and Cochrane CENTRAL covering January 2005 to February 2026. **Results**: Across published studies, DCBs have shown outcomes that are non-inferior to those of DES in selected ACS subsets, together with a lower risk of major bleeding attributable to shorter dual antiplatelet therapy (DAPT) requirements. Advances in intravascular imaging and lesion preparation, alongside emerging applications of artificial intelligence (AI) and robotic-assisted PCI, may further improve DCB performance, although evidence specific to DCB use in ACS remains limited for these adjunctive technologies. **Conclusions**: DCBs are a reasonable alternative to DES in selected patients with ACS, particularly those at high bleeding risk or with lesion subsets in which DES perform less well (small vessels, in-stent restenosis, bifurcations, diffuse disease). Adequately powered randomized trials with long-term follow-up are required before broader recommendations can be made.

## 1. Introduction

The therapeutic landscape for acute coronary syndrome (ACS) has been transformed by percutaneous coronary intervention (PCI). ACS encompasses a spectrum of coronary artery disease (CAD) presentations including ST-segment elevation myocardial infarction (STEMI), non-ST-segment elevation myocardial infarction (NSTEMI), and unstable angina (UA) [1]. Two additional clinical scenarios commonly intersect with ACS care: in-stent restenosis (ISR), the recurrent narrowing of a coronary artery within a previously implanted stent, and small-vessel disease (SVD), referring to atherosclerotic disease in coronary segments with reference vessel diameter ≤2.75–3.0 mm. Both ISR and SVD are well-established indications for drug-coated balloon (DCB) therapy, and growing evidence now extends DCB use into wider ACS populations.

For more than two decades, drug-eluting stents (DES) have served as the foundational treatment for ACS, reducing rates of target lesion revascularization (TLR) compared with bare-metal stents (BMS) [2]. However, permanent metallic scaffolds carry persistent long-term challenges, including late and very late stent thrombosis, neoatherosclerosis, and the need for prolonged dual antiplatelet therapy (DAPT) [3,4,5]. Extended DAPT, although necessary to prevent thrombotic events, increases the risk of major bleeding, particularly in older patients, those with comorbidities, and those receiving concomitant oral anticoagulation [4,6]. ACS lesions further complicate DES use because high thrombus burden and intense local inflammation may increase the risk of acute and subacute stent thrombosis [5].

DCBs are angioplasty balloons coated with an antiproliferative drug—most commonly paclitaxel or sirolimus—that is released to the vessel wall during a brief inflation, inhibiting neointimal hyperplasia without leaving a permanent implant [7,8]. Initially established for ISR and SVD, DCBs are increasingly used for de novo coronary lesions, including in ACS [9,10]. The “leave-nothing-behind” approach preserves native vessel integrity and vasomotion, simplifies subsequent revascularization, and allows for a shorter DAPT regimen, which is particularly relevant in ACS patients at high bleeding risk [6].

Before the development of DCBs, plain old balloon angioplasty (POBA) was the only balloon-based revascularization option. POBA produces higher rates of restenosis and repeat revascularization than stenting, However a clear mortality advantage of stents over POBA has not been established; the principal benefit of stenting lies in reducing repeat revascularization. POBA is now generally reserved for situations in which stenting is unsuitable. A long-term Swedish nationwide analysis confirmed that POBA is associated with higher rates of repeat revascularization than DES, providing a useful comparator for understanding the incremental benefit conferred by drug delivery alone with DCBs [11].

Recent state-of-the-art syntheses confirm that ACS management continues to evolve rapidly, with new evidence on antithrombotic strategies, complete revascularization, and intracoronary imaging shaping contemporary practice [1]. Within this evolving framework, this review summarizes contemporary evidence, practical methodology, and emerging adjunctive technologies relevant to DCB therapy in ACS, drawing on pivotal trials, registries, and meta-analyses available through early 2026.

## 2. Methodological Framework: Scope of This Narrative Review

This narrative review aims to provide a comprehensive overview of the evidence base for Drug-Coated Balloon (DCB) therapy in Acute Coronary Syndrome (ACS). While not a PRISMA-compliant systematic review, a structured approach was employed for article identification and selection.

### 2.1. Search Strategy

Relevant articles were identified through systematic searches of electronic databases including PubMed/MEDLINE, Embase, Scopus, Web of Science, and Cochrane Central Register of Controlled Trials (CENTRAL). The search covered the period from January 2005 to February 2026. The search strategy combined Medical Subject Headings (MeSH) terms and keywords related to DCBs and ACS. A representative search string included combinations of: (“drug-coated balloon” OR “drug-eluting balloon” OR “paclitaxel-coated balloon” OR “sirolimus-coated balloon”) AND (“acute coronary syndrome” OR “STEMI” OR “NSTEMI” OR “unstable angina” OR “myocardial infarction” OR “small-vessel disease” OR “in-stent restenosis” OR “de novo lesion”). Reference lists of retrieved articles, recent guideline documents (e.g., ESC guidelines), and consensus statements were also manually reviewed for additional relevant publications.

### 2.2. Inclusion and Exclusion Criteria

Inclusion Criteria: * Randomized controlled trials (RCTs) comparing DCB with Drug-Eluting Stents (DES) or other revascularization strategies in ACS patients. * Prospective and retrospective registries reporting outcomes of DCB use in ACS. * Meta-analyses and systematic reviews on DCB therapy in ACS. * Studies published in English.

Exclusion Criteria: * Case reports, editorials, letters, and review articles (unless they were comprehensive meta-analyses). * Studies focusing exclusively on peripheral artery disease or non-coronary applications of DCBs. * Non-peer-reviewed conference proceedings or abstracts without full publication. * Studies with fewer than 50 patients.

### 2.3. Study Selection and Data Synthesis

Articles were screened by title and abstract, followed by full-text review for eligibility. Priority was given to RCTs and large registries. Given the narrative nature of this review, no formal risk-of-bias assessment or quantitative synthesis (meta-analysis) was performed. Instead, findings are presented thematically, summarizing key outcomes and insights from the selected literature. Readers seeking pooled effect estimates are referred to existing systematic reviews and meta-analyses. The conclusions drawn reflect a balanced synthesis of the available evidence, acknowledging both the strengths and limitations of DCB therapy in various ACS subsets.

## 3. Mechanism of Action and Pharmacology

**Figure 1:**
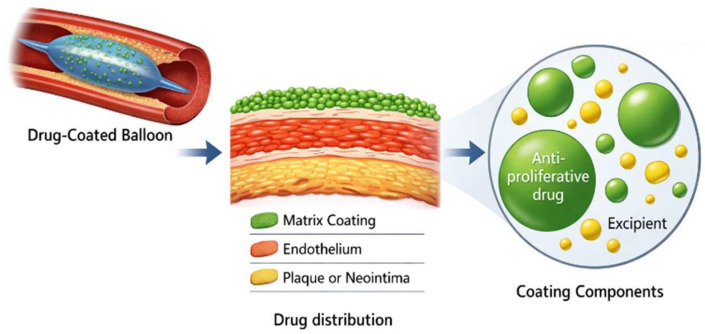
Mechanism of action of drug-coated balloon (created by the author).

DCB technology relies on the rapid transfer of a lipophilic antiproliferative drug from the balloon surface to the arterial wall during a brief inflation, typically 30 to 60 s in duration. Drug delivery and tissue retention are determined by the choice of drug, the excipient/coating matrix, and the surface architecture of the balloon [12] (Figure 1).

### 3.1. Drug Transfer Kinetics and Tissue Retention

The two predominant drug classes employed in DCB coatings are paclitaxel and sirolimus (or sirolimus analogues), each with distinct pharmacological properties.

Paclitaxel is a highly lipophilic cytotoxic agent that binds to tubulin, stabilizing microtubules, arresting the cell cycle in M phase, and inducing apoptosis of vascular smooth muscle cells, which are key contributors to neointimal hyperplasia [13,14]. Its lipophilicity supports rapid uptake into the arterial wall and prolonged tissue retention, often lasting weeks to months after a single transient application—properties central to its anti-restenotic effect [14].

Sirolimus (rapamycin) is a cytostatic macrolide that binds to FKBP-12 and inhibits the mammalian target of rapamycin (mTOR), causing G1-phase cell cycle arrest in vascular smooth muscle cells [15]. Sirolimus has been extensively used in DES, but its lower lipophilicity limited its use in early DCB platforms. Advances in nano-encapsulation and crystalline coating technologies have produced sirolimus-coated balloons (SCBs) with anti-restenotic efficacy comparable to paclitaxel-coated devices [16,17].

### 3.2. Excipients and Coating Technologies

Excipients are inert chemical additives that retain the drug on the balloon surface during navigation through the vasculature and facilitate uniform release and uptake into the vessel wall on inflation [18]. Excipient choice affects drug transfer efficiency, coating homogeneity, and bioavailability at the target site [19]. Commonly used excipients include iopromide (a contrast agent), urea, and lipid-based carriers; iopromide-based coatings achieve rapid release, while certain lipid formulations are designed for more sustained delivery.

The structure of the coating -crystalline versus amorphous- also influences performance. Crystalline paclitaxel coatings achieve greater tissue retention and more sustained anti-restenotic effects than amorphous formulations [20]. For sirolimus, micro-crystalline and nano-encapsulated formulations have improved drug solubility and stability, helping to overcome the pharmacokinetic limitations of sirolimus in a balloon-based delivery system [21]. These iterative refinements in coating chemistry continue to expand DCB applicability, although direct head-to-head comparisons of contemporary paclitaxel and sirolimus DCBs in ACS remain limited.

## 4. Practical Methodology and Lesion Preparation

**Figure 2:**
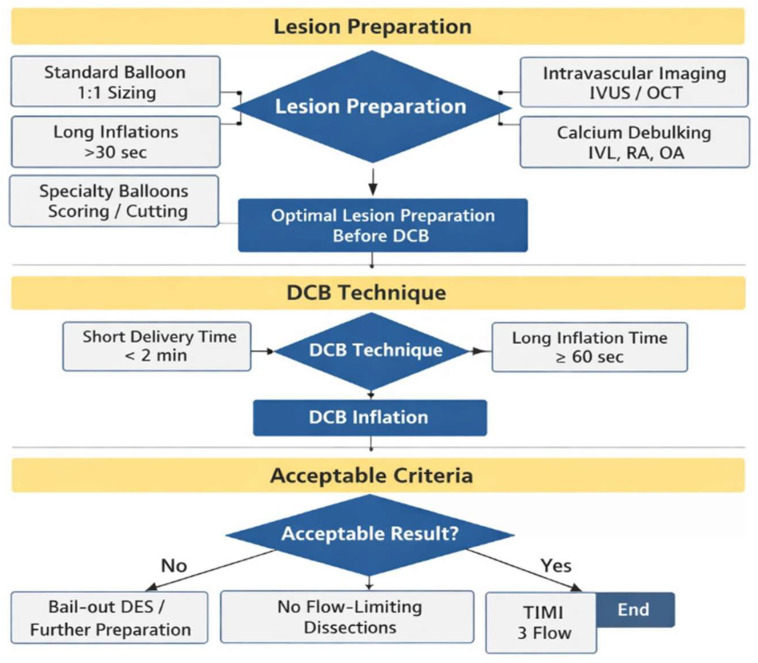
Recommended steps for optimal DCB outcome (created by the author).

Successful DCB therapy in ACS depends on careful procedural technique, with particular emphasis on lesion preparation and intracoronary imaging-guided assessment (Figure 2). The “leave-nothing-behind” strategy requires a deliberate approach to ensure adequate drug transfer and to minimize the need for bailout stenting [22].

### 4.1. Lesion Preparation

Optimal lesion preparation aims to achieve a residual stenosis ≤30% with no flow-limiting dissection (NHLBI grade C–F) after predilatation, providing a smooth and receptive vessel surface for drug uptake [23]. This is typically achieved with a non-compliant balloon sized at a 1:1 ratio to the distal reference vessel diameter, with prolonged inflation (>30 s) to allow plaque modification and to limit elastic recoil [24]. In fibrotic or moderately calcified lesions, scoring or cutting balloons can create controlled micro-dissections that improve vessel compliance and facilitate drug uptake [25]. For severely calcified lesions, which are common in ACS and frequently impede balloon expansion, adjunctive plaque modification with rotational atherectomy, orbital atherectomy, or intravascular lithotripsy (IVL) is often required [26]. IVL has gained particular interest because it modifies deep calcium with relatively low risk of vessel trauma [26].

### 4.2. Intravascular Imaging

Intravascular ultrasound (IVUS) and optical coherence tomography (OCT) are increasingly considered standard practice for DCB procedures, particularly for complex lesions and ACS [27]. These modalities provide real-time, high-resolution information on vessel size, plaque morphology, and the adequacy of lesion preparation that often cannot be obtained from angiography alone. For DCB procedures, IVUS and OCT play a crucial role in several aspects:

- Lesion Assessment: IVUS, with its superior penetration depth, is invaluable for assessing plaque burden, lumen area, and the extent and distribution of calcium, which is critical for planning optimal lesion preparation. OCT, offering superior spatial resolution (10–20 µm), provides detailed characterization of plaque composition (e.g., lipid-rich, fibrous, calcified) and precise measurement of lumen dimensions, guiding appropriate balloon sizing.
- Guidance for Lesion Preparation: Both IVUS and OCT help confirm the adequacy of predilatation, ensuring a smooth and receptive vessel surface for drug uptake. They can detect underexpanded segments or residual stenosis that might require further intervention before DCB inflation. For calcified lesions, IVUS and OCT can guide the use of adjunctive plaque modification tools like atherectomy or intravascular lithotripsy by precisely identifying calcium location and thickness.
- Detection of Dissections: OCT, in particular, excels at detecting subtle dissections that may influence DCB outcomes [28]. While severe dissections necessitate bailout stenting, recent data suggest that limited medial dissection visible on intravascular imaging before DCB inflation may be associated with favorable angiographic and clinical outcomes, possibly reflecting enhanced drug uptake [29]. This highlights the importance of imaging to differentiate between benign and flow-limiting dissections.
- Optimization of DCB Delivery: By providing real-time feedback on vessel morphology and lesion characteristics, IVUS and OCT help optimize DCB sizing and inflation, ensuring uniform drug transfer and minimizing the risk of complications.

**Figure 3:**
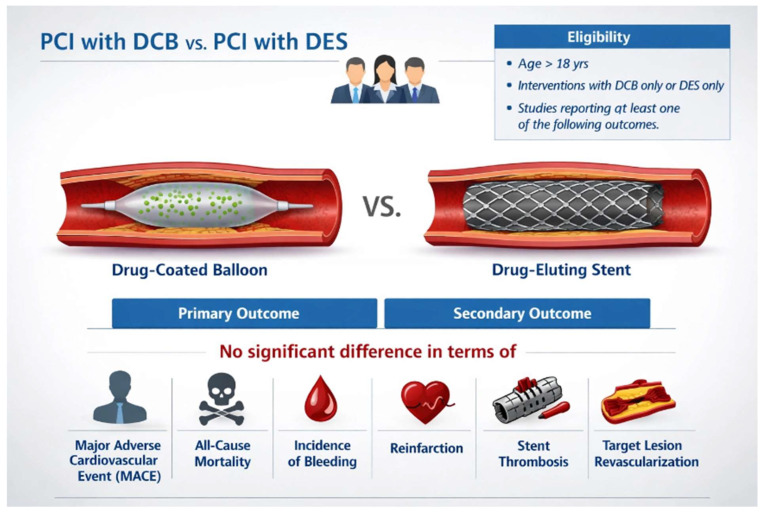
Drug-coated balloon versus drug-eluting stent (created by the author).

Overall, intravascular imaging enhances procedural success and helps to minimize the need for bailout stenting by ensuring optimal lesion preparation and precise DCB deployment. A schematic comparison of DCB and DES procedural and outcome features is shown in Figure 3.

### 4.3. Bailout Stenting

Despite optimal preparation, bailout stenting with a DES may be required when the DCB-only strategy fails to deliver an adequate angiographic result. Consensus criteria for bailout include flow-limiting dissection, significant residual stenosis, and persistent acute ischemia [22] (Table 1). The availability of a DES for bailout is therefore a mandatory component of any DCB-only strategy.

## 5. Clinical Evidence in ACS

The evidence base for DCBs in ACS has matured, with randomized controlled trials, registries, and meta-analyses now available across each major ACS subgroup. Because ACS is heterogeneous -encompassing STEMI, NSTEMI/UA, ACS occurring in small vessels, and ACS occurring at sites of prior in-stent restenosis- the strength and direction of evidence vary by setting and are summarized accordingly below. A summary of key trials is provided in Table 2.

### 5.1. ST-Segment Elevation Myocardial Infarction (STEMI)

Primary PCI for STEMI is performed in a clinical setting characterized by high thrombus burden, intense inflammation, and time pressure for reperfusion. DCB-only strategies have been investigated specifically in this population.

REVELATION was a randomized controlled trial that compared a paclitaxel-coated DCB strategy with a contemporary DES in 120 STEMI patients. The trial met its primary endpoint, demonstrating non-inferiority of the DCB approach for fractional flow reserve (FFR) at 9 months (0.92 ± 0.05 in the DCB group vs. 0.91 ± 0.06 in the DES group) [30]. Five-year follow-up data confirmed sustained non-inferiority for the composite endpoint of major adverse cardiac events (MACE) [31].

The UK STEMI Registry was a propensity-matched cohort study of 1139 STEMI patients which showed that DCB-only angioplasty was associated with rates of all-cause mortality and net adverse cardiac events comparable to those with second-generation DES, after a median follow-up exceeding three years [32].

The PAPPA pilot study was an earlier investigation of a DCB-only strategy in primary PCI for STEMI which established procedural feasibility and acceptable one-year outcomes, supporting the rationale for subsequent larger trials [33].

DEB-AMI, an earlier randomized trial that combined a paclitaxel DCB with a bare-metal stent, did not show benefit over bare-metal stent alone [34]. This neutral result has been attributed in part to early-generation DCB technology and the use of an obligatory bare-metal scaffold; subsequent DCB-only strategies using contemporary devices have produced more favorable results [44].

### 5.2. Non-ST-Segment Elevation Myocardial Infarction (NSTEMI) and Unstable Angina

NSTEMI patients tend to have heterogeneous lesion morphology and a higher prevalence of comorbidities than STEMI patients, factors that favor a tailored revascularization approach.

The PEPCAD NSTEMI trial randomized 210 NSTEMI patients to a DCB-only approach or stenting (BMS or DES) and demonstrated non-inferiority for target lesion failure (TLF) at 9 months (3.8% in the DCB group vs. 6.6% in the stent group) [35]. The trial supports a simpler DCB-only strategy in selected NSTEMI patients, particularly those with multivessel disease, diffuse lesions, or elevated bleeding risk.

### 5.3. ACS Occurring in Small-Vessel Disease

SVD remains one of the strongest established indications for DCBs, and the available trials include substantial proportions of patients presenting with ACS.

BASKET-SMALL 2 randomized 758 patients with SVD (28.2%, *n* = 214, with ACS at presentation) to a DCB or DES strategy. Although the overall primary endpoint of MACE at three years was non-inferior between groups, a prespecified subgroup analysis showed a significant interaction for cardiac death and non-fatal myocardial infarction at one year, with lower rates among ACS patients treated with DCB compared with DES [36,37]. This suggests a potential specific benefit in this high-risk subset that warrants confirmation in larger studies.

PICCOLETO II was a randomized trial in coronary SVD that demonstrated angiographic superiority of a paclitaxel DCB over an everolimus-eluting stent (EES), with significantly lower late lumen loss (LLL) and a reduction in adverse clinical events at three years [38].

### 5.4. ACS Occurring at Sites of In-Stent Restenosis

DCBs are a guideline-supported treatment option for ISR, and ISR not infrequently presents acutely as ACS.

The AGENT IDE trial provided contemporary evidence supporting paclitaxel-coated balloons for the treatment of ISR. The trial demonstrated superior TLF at one year (17.9% in the DCB group vs. 28.6% in the uncoated balloon group), met its primary endpoint, and supports DCB as a standard treatment for ISR [39]. Subgroup analyses suggest a consistent treatment effect in small-vessel ISR and multilayer ISR. Because ISR not infrequently presents as ACS, this evidence is directly relevant to the ACS population.

### 5.5. Recent Meta-Analyses and Registry Data

Meta-analyses and prospective registries have reinforced the role of DCBs across multiple settings. A 2024 meta-analysis pooling three-year outcomes from trials in coronary SVD concluded that DCBs are a reasonable and safe alternative to DES, with comparable efficacy [45]. A separate comprehensive review compared paclitaxel- and sirolimus-coated balloons, summarizing their pharmacodynamic differences and clinical performance [16]. The Eastbourne Registry, a prospective study of more than 2000 patients, demonstrated favorable safety and efficacy of a sirolimus-coated balloon across a wide range of clinical settings, including a substantial proportion of ACS patients [9,41]. The REC-CAGEFREE I trial, which compared DCB with primary stenting for de novo coronary lesions, provided additional evidence on DCB performance in broader indications [40]. The TRANSFORM II trial, a randomized comparison of a sirolimus-coated balloon with an everolimus-eluting stent in de novo native coronary vessels including larger vessels, is ongoing; its results are expected to clarify the role of SCBs in larger vessels and broader patient populations [42]. A recent bibliometric analysis confirms a marked increase in research output on DCB technology over the past decade, reflecting growing clinical interest [46].

### 5.6. Antithrombotic Therapy De-Escalation

The “leave-nothing-behind” strategy supported by the AGENT IDE trial provides an important biological foundation for considering antithrombotic therapy de-escalation in selected patients. Although AGENT IDE did not directly evaluate DAPT duration, its demonstration that paclitaxel-coated balloons are effective and safe for treating in-stent restenosis reinforces the advantages of avoiding additional metallic layers. By eliminating the need for further stent implantation, DCB therapy reduces the long-term risks associated with permanent scaffolds, such as late stent thrombosis, and may therefore support clinical pathways that favour shorter or simplified antithrombotic regimens in appropriate patient populations. This evidence strengthens the conceptual link between DCB-based ISR management and the potential for more individualised, lower-intensity antithrombotic strategies [39].

## 6. Strategies to Shorten DAPT in ACS Patients Treated with DCB

A central clinical advantage of a DCB-only strategy over DES is that an antiproliferative effect is delivered without leaving a permanent scaffold, removing the late stent thrombosis risk that has historically required prolonged DAPT [6]. This rationale has prompted several dedicated trials of abbreviated antiplatelet regimens in DCB-treated populations, although the supporting evidence base remains less mature than that for stent-related DAPT regimens.

REC-CAGEFREE II is an ongoing randomized clinical trial that compares 5-month ticagrelor monotherapy following only 1 month of DAPT against standard DAPT in ACS patients treated with a DCB strategy [43]. The trial is specifically designed to test whether the DCB-only approach can support markedly shorter DAPT durations without compromising ischemic safety in ACS, with the primary aim of reducing major bleeding events. Its results are expected to provide the most direct evidence on this question.

The EROSION study examined the strategy of antithrombotic therapy without stenting in selected ACS patients with intact fibrous cap and underlying plaque erosion [47]. Although not a DCB study, EROSION supports the broader concept that, in carefully selected ACS lesions, omission of a permanent scaffold can be feasible—providing mechanistic support for DCB-based and DCB-adjacent strategies that allow short-duration antiplatelet regimens.

Existing evidence from REVELATION, the UK STEMI Registry, and PEPCAD NSTEMI suggests that a 1- to 3-month DAPT course following DCB is safe in selected ACS patients, particularly those at high bleeding risk [30,32,35]. By comparison, contemporary guidelines recommend 6 to 12 months of DAPT following DES implantation in ACS, and longer durations in selected patients [6]. The reduced DAPT requirement after DCB is therefore a potentially decisive advantage in older patients, those with multiple comorbidities, those with prior bleeding, and those with a competing indication for oral anticoagulation. Adequately powered comparative trials with hard ischemic endpoints, however, are still required before short-DAPT-after-DCB regimens can be recommended as standard care across broader ACS populations.

## 7. Adjunctive Technologies: Artificial Intelligence and Robotics

Two adjunctive technologies—artificial intelligence (AI) image analysis and robotic-assisted PCI—have generated considerable interest in interventional cardiology and may eventually enhance the precision and reproducibility of DCB-based procedures. It is important to acknowledge, however, that direct prospective evidence specifically demonstrating improved DCB outcomes in ACS with these adjunctive technologies is currently lacking. Their role in DCB therapy should therefore be considered investigational rather than established.

### 7.1. Artificial Intelligence

**Figure 4:**
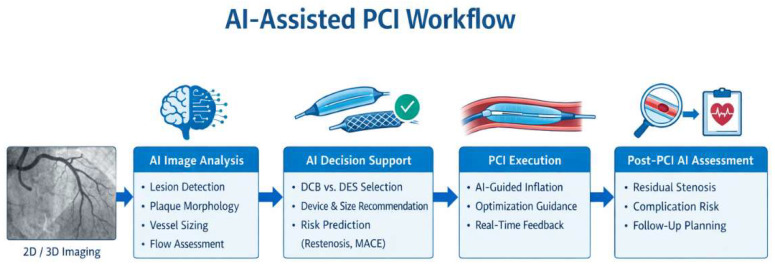
AI-assisted workflow during PCI (created by the author).

AI algorithms are being developed for use across the PCI workflow, with applications in pre-procedural planning, real-time intra-procedural guidance, and outcome prediction (Figure 4). In the context of DCB procedures, AI aims to:

- Enhance Lesion Assessment and Sizing: AI can automate the quantification of lumen dimensions, plaque morphology, and calcium burden from intracoronary imaging modalities such as IVUS and OCT. This can lead to more precise DCB sizing and selection, potentially improving drug transfer and reducing complications [48].
- Optimize Lesion Preparation: By identifying calcified or lipid-rich segments at higher risk of suboptimal DCB performance, AI-supported automated plaque characterization can guide adjunctive lesion preparation techniques, ensuring a more receptive vessel for DCB deployment [48].
- Decision Support: AI-driven decision-support tools could integrate imaging, physiological data (FFR, quantitative flow ratio [QFR]), and patient characteristics to help clinicians select appropriate revascularization strategies, including DCB-only versus stenting approaches [49,50].
- Outcome Prediction: AI models may predict procedural success and long-term outcomes, allowing for personalized treatment strategies.

However, dedicated outcomes evidence demonstrating that AI-guided DCB strategies improve clinical outcomes in ACS is currently lacking, and current AI tools have generally been validated in mixed PCI populations rather than in DCB-treated cohorts specifically.

### 7.2. Robotic-Assisted PCI

Robotic platforms such as the Corindus CorPath and Robocath R-One have been studied for routine PCI, with feasibility and safety initially established in studies such as PRECISE [51,52]. For DCB delivery, robotic assistance offers several potential advantages:

Enhanced Procedural Precision—Robotic PCI enables controlled, fine-increment movements of guidewires and devices, improving procedural precision and reducing operator-dependent variability.

Improved Operator Ergonomics—By allowing operators to perform PCI from a seated, radiation-shielded workstation rather than standing in heavy lead aprons, robotic systems improve ergonomics and reduce physical strain.

Significant Reduction in Operator Radiation Exposure—Relocating the operator to a protected console substantially reduces radiation exposure, enhancing occupational safety in the cath lab.

Consistent and Stable Device Manipulation—The robotic platform provides stable, reproducible control of interventional devices, supporting consistent guidewire and balloon positioning.

Demonstrated Safety and Feasibility—The PRECISE study confirmed that robotic PCI is safe and feasible, with high procedural success rates and no device-related complications.

However, direct prospective comparative evidence on robotic-assisted DCB delivery in ACS is currently lacking, and routine clinical use remains limited to centers with specific institutional experience and is not yet supported by outcome trials in ACS.

## 8. Advantages and Limitations of DCB Therapy in ACS

### 8.1. Advantages

Several features of a DCB-only approach are particularly relevant in the ACS setting (Table 3).

### 8.2. Limitations

DCB therapy has important limitations that constrain broader application. The principal acute-phase concern is the risk of vessel recoil and flow-limiting dissection after balloon inflation. Optimal results require careful lesion preparation and operator experience; suboptimal preparation may translate into inadequate drug transfer and increased rates of bailout stenting [22]. A related issue is the operator learning curve. Achieving reliable DCB outcomes requires a shift from a “stent-every-lesion” approach to one in which lesion preparation, predilatation adequacy, and intracoronary imaging-guided assessment are central [27]. This transition has implications for training and case volumes.

Evidence in larger coronary vessels (>3.5 mm) and in highly complex lesion subsets such as chronic total occlusions and severely calcified lesions remains limited, although TRANSFORM II and other ongoing studies are likely to address these gaps [42,56,57]. Geographic miss—failure to deliver drug across the entire lesion length, including margins—is a recognized concern with DCB and underscores the importance of accurate sizing and careful positioning [58]. Drug-specific concerns regarding paclitaxel safety in peripheral artery disease have prompted continued vigilance, although these signals have not been substantiated for coronary applications [39,45]. Sirolimus-coated balloons, being newer, require continued long-term follow-up across diverse populations.

## 9. Discussion

Drug-eluting stents have been the standard of care for ACS for more than two decades, and their limitations—late stent thrombosis, neoatherosclerosis, and the bleeding consequences of prolonged DAPT—provide the rationale for considering alternative strategies in selected patients. DCBs offer a “leave-nothing-behind” antiproliferative strategy that addresses several of these limitations.

The published evidence base supports DCB use in specific ACS subsets. but it is crucial to distinguish dedicated ACS trials from subgroup or extrapolated analyses. In STEMI, REVELATION demonstrated non-inferiority for FFR at 9 months and sustained non-inferiority for MACE at five years compared with DES [30,31]. In NSTEMI, PEPCAD NSTEMI demonstrated non-inferiority for TLF at nine months [35]. In ACS occurring in small vessels, the BASKET-SMALL 2 ACS subgroup analysis demonstrated lower rates of cardiac death and non-fatal MI at one year for DCB compared with DES [36,37]. However, BASKET-SMALL 2 primarily supports DCB use in small-vessel disease, and its ACS subgroup findings should not be interpreted as definitive evidence of generalized ACS benefit. Existing subgroup analyses overall suggest similar safety and efficacy between ACS and chronic coronary syndrome indications rather than a clear superiority signal in ACS In ACS occurring at sites of in-stent restenosis, AGENT IDE confirmed superior TLF for paclitaxel DCB compared with uncoated balloon [39]. By contrast, the earlier DEB-AMI trial did not demonstrate benefit, illustrating that early-generation DCB technology and DCB-plus-bare-metal-stent strategies are not interchangeable with contemporary DCB-only approaches; this underscores the importance of meticulous lesion selection, preparation, and procedural technique [34]. Recent state-of-the-art reviews of contemporary ACS care emphasize the importance of individualized antithrombotic and revascularization strategies, into which DCBs increasingly fit [1].

A key limitation of the current evidence base is the relative scarcity of long-term follow-up beyond 3 to 5 years. Although the available data are encouraging, longer-term durability of the anti-restenotic effect and detection of late safety signals require continued surveillance, and this lack of long-term data remains a meaningful constraint on broader recommendations. In addition, randomized DCB trials have generally been powered for composite or surrogate endpoints rather than for hard clinical endpoints such as cardiovascular mortality, recurrent myocardial infarction, or stroke considered individually. Claims of non-inferiority therefore rest on composite or angiographic endpoints; adequately powered trials with individual hard endpoints are still required to definitively establish equivalence with DES on these outcomes. Furthermore, greater emphasis must be placed on unresolved issues that complicate the routine use of DCBs in ACS. These include the variable and sometimes high rates of bailout stenting, inherent lesion-selection bias in many studies, the significant operator learning curve required for optimal lesion preparation, and the challenges posed by high thrombus burden in ACS. Other concerns such as vessel recoil, dissections, geographic miss, and the limited external validity of registry-based evidence further highlight the need for cautious interpretation of the current data.

The role of POBA also deserves comment. Stenting reduced the need for repeat revascularization compared with POBA, but a clear mortality advantage of stents over POBA was not demonstrated; the principal benefit of stenting lies in reducing TLR. POBA is therefore best regarded as a context-specific option in scenarios where stenting is contraindicated or technically unfeasible and DCB is unavailable, with the recognition that restenosis rates are higher than with DCB [11].

Economic considerations are also relevant. The unit cost of a DCB is comparable to or slightly higher than that of a DES, but the overall procedural cost may be increased by adjunctive lesion preparation tools (scoring or cutting balloons, IVL) and by routine use of intracoronary imaging. These up-front costs may be partly offset by reductions in long-term DAPT requirements, fewer bleeding complications, and lower TLR rates. Robust health-economic analyses comparing DCB and DES strategies across health systems are needed to inform reimbursement and policy decisions.

Finally, the integration of AI-assisted image analysis and robotic-assisted PCI may improve the precision of DCB delivery, but it should be re-emphasized that direct prospective evidence specifically demonstrating improved DCB outcomes with these adjunctive technologies in ACS is currently lacking. Their role should be considered investigational pending dedicated outcome data.

## 10. Conclusions

DCBs represent a safe and effective option for selected patients with ACS. They provide anti-restenotic efficacy comparable to DES across multiple ACS subsets, with the additional advantages of shorter DAPT requirements, preservation of native vessel integrity, and avoidance of permanent metallic implant complications. They are particularly well suited to high-bleeding-risk patients, ACS occurring in small vessels or at sites of in-stent restenosis, and selected complex lesion morphologies. Limitations include the need for meticulous lesion preparation and operator experience, the relative scarcity of long-term and hard-endpoint data, and the absence of dedicated outcomes evidence for AI- and robotics-assisted DCB delivery. Adequately powered randomized trials with longer follow-up—together with the awaited results of TRANSFORM II, REC-CAGEFREE II, and other ongoing studies—should help define the place of DCBs alongside DES in routine ACS care.

## Figures and Tables

**Table 1:** Bailout criteria for DCB-only strategy.

Significant Flow-Limiting Dissection	Residual Stenosis	Persistent Acute Ischemia
Any dissection classified as NHLBI Type C–F, or any dissection causing TIMI flow grade < 3, requires immediate stenting.	Residual stenosis > 50% by angiography, or inadequate lumen gain on intravascular imaging, despite repeat DCB inflation.	Ongoing chest pain, ST-segment changes on ECG, or hemodynamic instability suggestive of acute ischemia.

**Table 2:** Summary of key clinical trials evaluating DCBs in ACS.

Study	*N*	Population/Indication	Comparators	Main Results
REVELATION	120	STEMI	Paclitaxel DCB vs. DES	Non-inferior FFR at 9 months; non-inferior MACE at 5 years [30,31]
UK STEMI Registry	1139	STEMI (real-world, propensity-matched)	DCB-only vs. 2nd-generation DES	Comparable all-cause mortality and NACE at >3 years [32]
PAPPA pilot	100	STEMI	DCB-only (single arm)	Procedural feasibility; acceptable 1-year outcomes [33]
DEB-AMI	150	STEMI	DCB + BMS vs. BMS alone vs. DES	No benefit of DCB + BMS over BMS [34]
PEPCAD NSTEMI	210	NSTEMI	DCB-only vs. BMS or DES	Non-inferior TLF at 9 months (3.8% vs. 6.6%) [35]
BASKET-SMALL 2	758 (214 ACS)	SVD (ACS subgroup)	Paclitaxel DCB vs. DES	Non-inferior MACE at 3 years; lower cardiac death/non-fatal MI at 1 year in ACS subgroup [36,37]
PICCOLETO II	232	SVD	Paclitaxel DCB vs. EES	Lower LLL; reduction in adverse clinical events at 3 years [38]
AGENT IDE	600	ISR	Paclitaxel DCB vs. uncoated balloon	Superior TLF at 1 year (17.9% vs. 28.6%) [39]
REC-CAGEFREE I	2272	De novo coronary lesions	Paclitaxel DCB vs. primary stenting	Evidence on DCB performance in broader de novo indications [40]
Eastbourne Registry	~2000	All-comers (incl. ACS)	Sirolimus DCB (single arm)	Favorable real-world safety and efficacy [9,41]
TRANSFORM II	Ongoing	De novo native coronary vessels (incl. larger vessels)	Sirolimus DCB vs. EES	Awaited; results expected to inform DCB use in larger vessels [42]
REC-CAGEFREE II	Ongoing	ACS treated with DCB	5-month ticagrelor mono after 1-month DAPT vs. standard DAPT	Awaited; tests abbreviated DAPT after DCB-only ACS PCI [43]

BMS = bare-metal stent; DCB = drug-coated balloon; DES = drug-eluting stent; EES = everolimus-eluting stent; FFR = fractional flow reserve; ISR = in-stent restenosis; LLL = late lumen loss; MACE = major adverse cardiac events; NACE = net adverse cardiac events; NSTEMI = non-ST-segment elevation myocardial infarction; STEMI = ST-segment elevation myocardial infarction; SVD = small-vessel disease; TLF = target lesion failure.

**Table 3:** Principal advantages of a DCB-only strategy in ACS.

Advantage	Mechanism/Clinical Relevance
Significant reduction in bleeding risk	Allows shorter DAPT (1–3 months vs. 6–12 months for DES); particularly relevant in older patients, those with comorbidities, or those on oral anticoagulation [6,30,39,43].
Preservation of native vessel integrity and function	Permits positive vessel remodeling, preserves endothelial function and physiological vasomotion, and leaves future revascularization options unimpeded by overlapping metal layers [32,33,53].
Simplified treatment of complex lesions	Effective in bifurcation lesions (avoids jailing the side branch and complex two-stent techniques), in SVD (where DES carry a relatively larger metal-to-vessel ratio), and across diffuse disease (avoiding multiple overlapping stents and stent fracture risk) [35,36,38,54].
Avoidance of stent-related complications	No late or very late stent thrombosis, neoatherosclerosis, or chronic inflammatory response to permanent struts [55].

## Data Availability

No new data were created or analyzed in this study.
